# Metabolomic Biomarkers of Multiple Sclerosis: A Systematic Review

**DOI:** 10.3389/fmolb.2020.574133

**Published:** 2020-12-14

**Authors:** Lachlan Porter, Alireza Shoushtarizadeh, George A. Jelinek, Chelsea R. Brown, Chai K. Lim, Alysha M. de Livera, Kelly R. Jacobs, Tracey J. Weiland

**Affiliations:** ^1^Neuroepidemiology Unit, Melbourne School of Population and Global Health, The University of Melbourne, Carlton, VIC, Australia; ^2^The Peter Doherty Institute for Infection and Immunity, Melbourne, VIC, Australia; ^3^Department of Biomedical Sciences, Faculty of Medicine and Health Sciences, Macquarie University, Macquarie Park, NSW, Australia

**Keywords:** metabolomics, MS, multiple sclerosis, multiple sclerois and neuroimmunology, systematic (literature) review, biomarker, neuroimmunological disease, metabolomics (OMICS)

## Abstract

**Background:**

Magnetic resonance imaging (MRI), cerebrospinal fluid (CSF) analysis, and the McDonald’s clinical criteria are currently utilized tools in diagnosing multiple sclerosis. However, a more conclusive, consistent, and efficient way of diagnosing multiple sclerosis (MS) is yet to be discovered. A potential biomarker, discovered using advances in high-throughput sequencing such as nuclear magnetic resonance (NMR) spectroscopy and other “Omics”-based techniques, may make diagnosis and prognosis more reliable resulting in a more personalized and targeted treatment regime and improved outcomes. The aim of this review was to systematically search the literature for potential biomarkers from any bodily fluid that could consistently and accurately diagnose MS and/or indicate disease progression.

**Methods:**

A systematic literature review of EMBASE, PubMed (MEDLINE), The Cochrane Library, and CINAHL databases produced over a thousand potential studies. Inclusion criteria stated studies with potential biomarker outcomes for people with MS were to be included in the review. Studies were limited to those with human participants who had a clinically defined diagnosis of MS and published in English, with no limit placed on date of publication or the type of bodily fluid sampled.

**Results:**

A total of 1,805 studies were recorded from the literature search. A total of 1,760 studies were removed based on their abstract, with a further 18 removed after considering the full text. A total of 30 studies were considered relevant and had their data retrieved and analyzed. Due to the heterogeneity of focus and results from the refined studies, a narrative synthesis was favored.

**Conclusion:**

Several promising candidate biomarkers suitable for clinical application in MS have been studied. It is recommended follow-up studies with larger sample sizes be completed on several potential biomarkers.

## Background

Multiple sclerosis (MS) is a chronic, progressive, neurological disease which affects over 2.5 million people worldwide (Raphael et al., 2015). Disease presentation and progression vary between patients, making diagnosis sometimes challenging. MS is often categorized into three principal subtypes: relapsing remitting MS (RRMS), secondary progressive MS (SPMS), and primary progressive MS (PPMS). RRMS is defined as having episodes of relapses or attacks where new symptoms appear, followed by periods of remission where there are little to no symptoms, although neurological damage accumulates with repeated relapses. SPMS is characterized by gradual worsening after an initial relapsing disease course, with or without acute exacerbations during the progressive course (Lublin et al., 2014). PPMS is defined as continued worsening without exacerbations prior to clinical progression (Lublin et al., 2014). Clinically isolated syndrome (CIS) is the term given to the first clinical onset of potential MS where inflammatory demyelination has occurred but the criterion of dissemination has not been fulfilled (EfendI, 2015).

At present, MS is diagnosed using clinical tests such as the McDonald criteria (McDonald et al., 2001), magnetic resonance imaging (MRI), and the presence of oligoclonal bands (OCB) in cerebrospinal fluid (CSF) (Raphael et al., 2015). The discovery of a molecular biomarker that could assist with the diagnosis of MS would allow treatment and management to begin sooner. A molecular biomarker could also be used to quantify disease exacerbation and assess response to treatment.

Traditionally, two techniques are used to search for biomarkers: hypothesis-based and discovery-based methods (McDermott et al., 2013). Hypothesis-based biomarker searches are focused on understanding disease mechanisms, to date not fully elucidated in MS. Discovery-based biomarker methods seek to identify changes in the concentration of molecular species, such as metabolites, that are associated with the disease of interest (McDermott et al., 2013).

Metabolites are defined as low molecular weight (<900 Da) organic and inorganic molecules which are the reactants, intermediates, or products of enzyme-mediated biochemical reactions (Dunn et al., 2011). The compositional diversity of metabolites results in a range of physiochemical properties, making their investigation a challenge. Analytical chemistry techniques such as mass spectrometry and nuclear magnetic resonance (NMR) spectroscopy which may be coupled with chromatography are the most common techniques used to identify and quantify metabolites (Dunn et al., 2011). An explosion in these high-throughput sequencing and “-omics”-based technologies over the past decade has been the catalyst for many published candidate biomarkers for MS; however, many of these are general inflammatory markers and have not yet translated into practical, clinical biomarkers (Housley et al., 2015). Another problem currently faced when using high-throughput sequencing on biological samples from people with MS is the considerable difference in metabolite concentrations between studies. It has been hypothesized that this is due to MS disease heterogeneity as well as technical and chemometric limitations (Reinke et al., 2014) and could also be due to variations in sample cohorts and differences in classification criteria.

The aim of this review was to systematically collate published literature on potential biomarkers found in the biological samples collected from individuals with confirmed MS.

## Methods

### Literature Search Strategy

The review was registered with Prospero (CRD42017060866) at https://www.crd.york.ac.uk/prospero/prior to the official systematic search being conducted on the 30th of September 2017. Databases searched included EMBASE, PubMed (MEDLINE), The Cochrane Library, and CINAHL using the search strategy that can be found at *https://www.crd.york.ac.uk/PROSPEROFILES/60866_STRATEGY_20170728.pdf*. Medical subject heading (MeSH) terms were used where applicable. Articles were imported into EndNote and duplicates removed electronically using EndNote software and checked manually before being screened based on title and abstract for relevance against the inclusion/exclusion criteria. Additional searches of reference lists of relevant papers and gray literature were undertaken. Full-length papers of abstracts that matched the inclusion/exclusion criteria were retrieved for further assessment of relevance. To minimize selection bias and ensure accuracy, two independent researchers (LP and CB) screened full-length papers for relevancy. A third, independent, blinded researcher (AS) settled disputes. AS and LP screened the full texts of the refined papers through consensus for relevance according to the inclusion/exclusion criteria. The process of refining studies is reported following the Preferred Reporting Items for Systematic Reviews and Meta-Analyses (PRISMA) flow diagram from the PRISMA statement (Moher et al., 2009).

### Inclusion Criteria

Studies of the metabolomic profiles or studies with potential biomarker outcomes for people diagnosed with MS were included in the review. An *a priori* decision was made to include lipids of a low atomic weight and metabolites as relevant to the metabolomic profile (Dunn et al., 2011). Studies were limited to those with human participants and published in English, with no limit placed on the year of publication. Studies were not excluded based on the type of bodily fluid sampled but were excluded if the participants lacked a clinical diagnosis of MS. Systematic reviews and meta-analyses were also excluded from the review. After reviewing the abstracts, the protocol was updated so that studies focusing on “lipid profiles” were also excluded. We defined “lipid profiles” to include cholesterol (both high and low density lipoprotein) and triglycerides. This decision was made after reviewing the abstracts due to the excessive number of eligible studies found. A review of lipid profiles will be reported elsewhere in a planned separate paper.

### Data Extraction and Analysis

Two researchers (LP and AS) extracted data independently from the refined papers (Tables 1, 2). Due to the heterogeneity of focus and results from the refined studies, it was not appropriate to conduct a meta-analysis, so a narrative synthesis was performed. The data points taken from each study included year of publication, sample size, study design, control used, sample type, sample analysis technique, metabolite pathway(s) examined, and the main findings from the metabolites/potential biomarker analyzed (in quantitative form where possible). It was assumed that if the study did not report blinding then the researchers were not blinded. Authors were not contacted to provide further information. The Quadomics criteria were used in the quality assessment of the included papers. Quadomics is a set of 16 criteria that have been developed to assess the quality of -omics-based studies. The tool is an adaption of the widely used QUADAS tool—an evidence-based quality assessment tool to be used in systematic reviews of diagnostic accuracy studies—adding additional criteria to check the collection and handling of the differing biological samples used in -omics research.

**TABLE 1:** Key data points extracted from the refined studies.

Author	Title	Year of publication	Sample size	Study design	Type of control	Sample analysis technique
Aasly et al.	Cerebrospinal fluid lactate and glutamine are reduced in multiple sclerosis	1997	10 patients with chronic, progressive MS • 10 RRMS • 14 HC	Cross-sectional	HC	MRS
Aeinehband et al.	Cerebrospinal fluid kynurenine in multiple sclerosis; relation to disease course and neurocognitive symptoms	2016	Cohort 1 • 38 MS patients • 20 patients with “other neurological disorder” (OND) • 13 patients with other, inflammatory neurological disorders (iOND) • Cohort 2 • 48 RRMS	Cross-sectional	OND	LC-MS
Augutis et al.	Cerebrospinal fluid biomarkers of amyloid metabolism in multiple sclerosis	2013	54 RRMS • 33 SPMS • 28 HC	Cross-sectional and longitudinal	HC and baseline samples	Immunoassays, immunoprecipitation, mass spectrometry, LC-MS
Bystricka et al.	Methionine metabolism and multiple sclerosis	2017	37 RRMS • 8 SPMS • 37 HC	Cross-sectional	HC	ELISA assays, chromatography
Checa et al.	Hexosylceramides as intrathecal markers of worsening disability in multiple sclerosis	2015	41 RRMS • 15 SPMS • 9 PPMS • 13 clinical isolated symptoms (CIS) • Two control groups: • 38 OND • 18 iOND. • 17 of the RRMS patients involved in the longitudinal study	Cross-sectional and longitudinal (involving 17 RRMS patients with multiple measurements taken 4 years apart)	OND, iOND and baseline samples for the longitudinal study	ELISA assays, lLC-ESI-MS
Dickens et al.	A type 2 biomarker separates relapsing–remitting from secondary progressive multiple sclerosis	2014	Cohort A • 17 PPMS • 46 SPMS • 22 RRMS • 14 controls. • Cohort B • 10 SPMS • 6 RRMS • 7 controls • Cohort C • 10 SPMS • 5 RRMS • 7 controls	Cross-sectional	“Control”	NMR spectroscopy
Cocco E	1H-NMR analysis provides a metabolomic profile of patients with multiple sclerosis	2015	61 RRMS • 12 patients with “progressive” MS • 88 HC	Cross-sectional	HC	1H-NMR spectroscopy
Gebregiworgis et al.	A Urinary Metabolic Signature for Multiple Sclerosis and Neuromyelitis Optica	2016	8 RRMS • 9 neuromyelitis optica (NMO) • 7 HC	Cross-sectional	HC and NMO	1H-NMR spectroscopy
Hon et al.	Membrane saturated fatty acids and disease progression in Multiple Sclerosis patients	2009	28 RRMS • 1 PPMS • 2 SPMS • 30 HC	Cross-sectional	HC	GC
Hon et al.	Plasma non-esterified fatty acids in patients with multiple sclerosis	2011	31 MS • 30 HC	Cross-sectional	HC	GC
Hon et al.	Erythrocyte membrane fatty acids in patients with multiple sclerosis	2009	28 RRMS • 1 PPMS • 2 SPMS • 30 HC	Cross-sectional	HC	GC
Kim et al.	Metabolomic profiling of CSF in multiple sclerosis and neuromyelitis optica spectrum disorder by nuclear magnetic resonance	2017	50 MS • 57 patients with NMO • 17 HC	Cross-sectional	HC	NMR
Lazzarino et al.	Cerebrospinal fluid ATP metabolites in multiple sclerosis	2010	21 MS	Prospective, longitudinal study where previous samples had been taken 3 years apart	Baseline levels and data from controls of a previous study	HPLC and ELISA
Lim et al.	Kynurenine pathway metabolomics predicts and provides mechanistic insight into multiple sclerosis progression	2017	Cohort 1 • 50 RRMS • 20 SPMS • 17 PPMS • 49 HC • Cohort 2 • 44 RRMS • 15 SPMS • Cohort 3 • 10 RRMS • 20 SPMS • 6 HC	Cross-sectional and longitudinal	HC and baseline values	UHPLC and GC/MS
Lotsch et al.	Machine-Learned Data Structures of Lipid Marker Serum Concentrations in Multiple Sclerosis Patients Differ from Those in Healthy Subjects	2017	102 MS • 301 HC	Case–control	HC	LC-ESI-MS
Moussallieh et al.	Serum analysis by 1H Nuclear Magnetic Resonance spectroscopy: a new tool for distinguishing neuromyelitis optica from multiple sclerosis	2014	47 RRMS • 44 NMO • 42 HC	Cross-sectional	HC and NMO	1H-NMR spectroscopy
Moyano et al.	Levels of plasma sulfatides C18: 0 and C24: 1 correlate with disease status in relapsing-remitting multiple sclerosis	2013	14 RRMS • 14 HC	Cross-sectional	HC	UHPLC-MS/MS
Navarro and Segura	Plasma lipids and their fatty acid composition in multiple sclerosis	1988	61 MS (51 definite, 9 probable and 1 possible according to the classification by Rose et al.) • 61 HC	Cross-sectional	HC	Thin-layer chromatography for lipids, GC for fatty acids and enzymatic methods
Park et al.	Disease Type- and Status-Specific Alteration of CSF Metabolome Coordinated with Clinical Parameters in Inflammatory Demyelinating Diseases of CNS	2016	54 MS patients • 12 HC	Cross-sectional	HC	GC/MS
Pieragostino et al.	An integrated metabolomics approach for the research of new cerebrospinal fluid biomarkers of multiple sclerosis	2015	13 RRMS • 12 OND	Cross-sectional	OND	targeted MALDI-TOF-MS and untargeted LC-MS/MS
Poddighe et al.	Metabolomic analysis identifies altered metabolic pathways in Multiple Sclerosis	2017	28 RRMS • 4 progress MS • 33 HC	Cross-sectional	HC	GC/MS
Regenold et al.	Cerebrospinal fluid evidence of increased extra-mitochondrial glucose metabolism implicates mitochondrial dysfunction in multiple sclerosis disease progression	2008	22 remitted RRMS • 9 relapsed RRMS • 37 stationary SPMS • 17 relapsed SPMS • 18 HC	Cross-sectional, pilot	HC	GC/MS
Reinke et al.	Metabolomic profiling in multiple sclerosis: insights into biomarkers and pathogene	2014	11 RRMS • 3 SPMS • 1 CIS • 17 non-MS controls	Cross-sectional	Non-MS controls	1H-NMR spectroscopy
Salemi et al.	Blood lipids, homocysteine, stress factors, and vitamins in clinically stable multiple sclerosis patients	2010	28 RRMS • 12 SPMS • 80 HC (2 controls:1 MS case	Cross-sectional	HC	Enzymatic colorimetric tests HPLC and fluorimetry competitive magnetic separation
Sinclair et al.	NMR-based metabolomic analysis of cerebrospinal fluid and serum in neurological diseases—a diagnostic tool?	2010	Cohort 1 • 12 CSF and 11 serum samples from people with MS, • 25 CSF and 17 serum samples from people with IIH, • 9 CSF and 9 serum samples from people with CVD and • 41 CSF samples and 35 serum samples from an “other group” of diseases. • Cohort 2 had • 8 IIH • 3 MS • 14 fitting the “other” group	Cross-sectional	IIH and “other diseases”	1H-NMR spectroscopy
Sternberg et al.	Plasma pentosidine: a potential biomarker in the management of multiple sclerosis	2011	63 RRMS • 21 SPMS • 8 PPMS • 6 CIS • 43 HC	Cross-sectional	HC	HPLC
Vegara et al.	A lipidomic approach to the study of human CD4T lymphocytes in multiple sclerosis	2015	8 RRMS • 5 HC	Cross-sectional	HC	GC and MALDI-TOF MS

**TABLE 2:** Analytes extracted from refined studies, their concentration compared to their reference group, and other findings.

Classes/analytes	Biological fluid	Ref group	Higher or lower compared to ref group?	Other finding/comments
**Fatty acid and lipid metabolism and derivatives**				
Hexosylceramides (16:0, 24:1)	CSF (Checa et al., 2015)	OND, iOND, and baseline	RRMS: higher	Hexosylceramides (16:0, 24:1) were increased 4.7 years after baseline taken. 16:0 correlated with EDSS of patients
			Progressive MS: higher	
Phosphocholine	Serum (Dickens et al., 2014)	RRMS	SPMS: decreased	
Glycosphingolipids				
C18:0/C24:1 sulfatides	Plasma	HC		Positive correlation between ratio and EDSS in patients with RRMS
C16:0/C24:0 sulfatides	Plasma (Moyano et al., 2013)	HC		Positive correlation between ratio of sulfatides and time since last relapse
C16:0/C18:0 sulfatides	Plasma (Moyano et al., 2013)	HC		Positive correlation between ratio and age of RRMS patients
C16:0	Plasma (Hon et al., 2011)	HC	MS: increased	
	Plasma (Navarro and Segura, 1988)	HC	MS: increased	
	Plasma (Moyano et al., 2013)	HC	MS: no difference	
C18:0	Plasma (Hon et al., 2011)	HC	MS: increased	
	Plasma (Navarro and Segura, 1988)	HC	MS: decreased	
	Plasma (Moyano et al., 2013)	HC	MS: no difference	
C18:2 (linoleic acid, a polyunsat omega-3-FA)	Plasma (Navarro and Segura, 1988)	HC	MS: decreased	The decrease was correlated with EDSS
C20:4 (arachidonic acid)	Plasma (Navarro and Segura, 1988)	HC	MS: decreased	
C16:1—subgroup uncertain	Plasma (Navarro and Segura, 1988)	HC	MS: increased	
C20:0—subgroup uncertain	Plasma (Navarro and Segura, 1988)	HC	MS: increased	
C24:0—subgroup uncertain	Plasma (Moyano et al., 2013)	HC	RRMS: no difference	
C24:1—subgroup uncertain	Plasma (Moyano et al., 2013)	HC	RRMS: no difference	
	Plasma (Navarro and Segura, 1988)	HC	MS: increased	
27-Hydroxycholesterol	Plasma (Narayanaswamy et al., 2015)	HC	MS: decreased	HC > MS > OND
	Plasma (Narayanaswamy et al., 2015)	OND	MS: increased	
7a-Hydroxycholesterol	Plasma (Narayanaswamy et al., 2015)	HC	MS: decreased	
SM C18:2n-6	Plasma (Hon et al., 2011)	HC	MS: decreased	
NEFAs	Plasma (Hon et al., 2011)	HC	MS: increased	
C18:2n-6	Plasma (Hon et al., 2011)	HC	MS: increased	
C20:4n-6	Plasma (Hon et al., 2011)	HC	MS: increased	
C16:1n-7	Plasma (Hon et al., 2011)	HC	MS: increased	
C18:1n-7	Plasma (Hon et al., 2011)	HC	MS: increased	
C18:1n-9	Plasma (Hon et al., 2011)	HC	MS: increased	
C14:0	Plasma (Hon et al., 2011)	HC	MS: increased	
Fatty acid (refer to Dicken et al.)	Serum (Dickens et al., 2014)	RRMS	SPMS: decreased	
Glycerol	Serum (Cocco et al., 2015)	HC	MS: decreased	
3-Hydroxybutyrate (b-hydroxybutyric acid)	Urine (Gebregiworgis et al., 2016)	HC and NMO	MS: increased	
Acetoacetate	Serum (Cocco et al., 2015)	HC	MS: increased	
Acetone	Serum (Cocco et al., 2015)	HC	MS: increased	
1-Monopalmitin	CSF (Park et al., 2016)	HC	MS: increased	
1-Monostearin	CSF (Park et al., 2016)	HC	MS: increased	
PC C20:4n-6	Venous Blood (Hon et al., 2009b)	HC	MS: decreased	Levels are inversely correlated with EDSS
HDL-Cholesterol	Serum (Salemi et al., 2010)	HC	MS: increased	
Phosphatidylinositol	Serum (Vergara et al., 2015)	HC	RRMS: increased	Lipid species m/z 861,640 (could have been phosphatidylglycerol)
CL 72:8	Serum (Vergara et al., 2015)	HC	RRMS: increased	
CL 74:10	Serum (Vergara et al., 2015)	HC	RRMS: increased	
C18:1n-11	Serum (Vergara et al., 2015)	HC	RRMS: increased	
C18:3n-6	Serum (Vergara et al., 2015)	HC	RRMS: decreased	
C20:4n-6	Serum (Vergara et al., 2015)	HC	RRMS: increased	
Sat-FA	Serum (Vergara et al., 2015)	HC	RRMS: decreased	
MonoUnsat-FA	Serum (Vergara et al., 2015)	HC	RRMS: increased	
PolyUnsat-FA	Serum (Vergara et al., 2015)	HC	RRMS increased	
LysoPC (16:0, 18:0, 18:1)	Serum (Del Boccio et al., 2011)	HC	RRMS: decreased	
LysoPE (24:1/0:0)	Serum (Del Boccio et al., 2011)	HC	RRMS: increased	
15-Hydroxyeico-satetraenoic acid	CSF (Pruss et al., 2013)	Less active MS	Active MS: increased	
PGE	CSF (Pruss et al., 2013)	Less active MS	Active MS: increased	
Resolvin D1 (DHA-derived)	CSF (Pruss et al., 2013)	Active MS	Active MS: increased	
Pentadecanoic acid	CSF (Park et al., 2016)	HC	MS: increased	HC < remitting MS < relapsing MS
Oleic acid	CSF (Park et al., 2016)	HC	MS: increased	HC < remitting MS < relapsing MS
**Amino acid metabolism and derivatives**				
L-Asparagine	Plasma (Poddighe et al., 2017)	HC	MS: increased	
L-Ornithine	Plasma (Poddighe et al., 2017)	HC	MS: increased	
L-Glutamate	Plasma (Poddighe et al., 2017)	HC	MS: increased	
	CSF (Sinclair et al., 2010)	IIH and “other diseases”	MS: increased	
	CSF (Pieragostino et al., 2015)	HC	MS: increased	
	Serum (Moussallieh et al., 2014)	NMO	MS: decreased	
L-Glutamine	CSF (Lim et al., 2017)	HC	MS: decreased	RRMS < CPMS
	Plasma (Poddighe et al., 2017)	HC	MS: increased	
	Serum (Moussallieh et al., 2014)	HC	MS: decreased	MS > NMO
Pyroglutamate	CSF (Kim et al., 2017)	HC	MS: increased	
	Plasma (Poddighe et al., 2017)	HC	MS: decreased	
Methionine	Serum (Bystricka et al., 2017)	HC	SPMS: decreased	
			RRMS: decreased	
	CSF (Park et al., 2016)	HC	MS: increased	
Glutathione	Serum (Bystricka et al., 2017)	HC	SPMS: decreased	
			RRMS: decreased	
Tryptophan	Serum (Cocco et al., 2015)	HC	MS: decreased	
	CSF (Aeinehband et al., 2016)	OND	SPMS: decreased	
5-Hydroxytryptophan	Serum (Cocco et al., 2015)	HC	MS: decreased	
Quinolinic acid	Serum (Lim et al., 2017)	HC	PPMS: increased	PPMS > SPMS > RRMS > HC
			SPMS: increased	
Kynurenic acid	Serum (Lim et al., 2017)	HC	RRMS: increased	
			PPMS: decreased	
			SPMS: decreased	
Picolinic acid	Serum (Lim et al., 2017)	HC	RRMS: increased	
			PPMS: decreased	
			SPMS: decreased	
3-Hydroxykynurenine	Serum (Lim et al., 2017)	HC	PPMS: increased	PPMS > SPMS
			SPMS: increased	
QA/KA	Serum (Lim et al., 2017)	HC	PPMS: increased	PPMS > SPMS
			SPSM: increased	
	CSF (Aeinehband et al., 2016)	Remitting RRMS	Relapsing RRMS: increased	
KA/KYN	CSF (Aeinehband et al., 2016)	OND	PPMS: increased	
			SPMS: decreased	
Alanine	Serum (Cocco et al., 2015)	HC	MS: increased	
	CSF (Sinclair et al., 2010)	IIH and “other diseases”	MS: decreased	
Lysine	Serum (Moussallieh et al., 2014)	NMO	MS: decreased	
	Serum (Moussallieh et al., 2014)	HC	MS: increased	
Valine	CSF (Park et al., 2016)	HC	MS: increased	
	CSF (Kim et al., 2017)	Remitting MS	Relapsing MS: decreased	
	Serum (Moussallieh et al., 2014)	HC	MS: decreased	
Homocysteine	Serum (Salemi et al., 2010)	HC	MS: increased	
Isoleucine	CSF (Park et al., 2016)	HC	MS: increased	
	CSF (Kim et al., 2017)	Remitting MS	Relapsing MS: decreased	
Phenylalanine	CSF (Park et al., 2016)	HC	MS: increased	
	CSF (Reinke et al., 2014)	Non-MS Control	MS: decreased	
Tyrosine	CSF (Park et al., 2016)	HC	MS: increased	
Leucine	CSF (Park et al., 2016)	HC	MS: increased	
Proline	CSF (Park et al., 2016)	HC	MS: increased	
Putrescine	CSF (Park et al., 2016)	HC	MS: increased	HC < remitting MS < relapsing MS
Oxoproline (pyro-glutamate/glutamic acid)	CSF (Park et al., 2016)	HC	MS: increased	HC < remitting MS < relapsing MS
**Carbohydrate metabolism and derivatives**				
Pentosidine (derive from ribose)	Plasma (Sternberg et al., 2011)	HC	MS: increased	
Fructose	Plasma (Poddighe et al., 2017)	HC	MS: decreased	
	CSF (Regenold et al., 2008)	HC	RRMS: increased	
			SPMS: increased	
Myo-inositol	CSF (Reinke et al., 2014)	Non-MS controls	MS: increased	
	Plasma (Poddighe et al., 2017)	HC	MS: decreased	
Scyllo-inositol	Serum (Moussallieh et al., 2014)	NMO	MS: increased	
Threose	CSF (Park et al., 2016)	HC	MS: increased	
Threonate (threonic acid)	Plasma (Poddighe et al., 2017)	HC	MS: decreased	
	CSF (Reinke et al., 2014)	Non-MS controls	MS: increased	
Glucose	Serum (Cocco et al., 2015)	HC	MS: decreased	
	Serum (Dickens et al., 2014)	RRMS	SPMS: decreased	
	CSF (Kim et al., 2017)	HC and NMO	MS: decreased	
Lactate	CSF (Lim et al., 2017)	HC	MS: decreased	
	CSF (Regenold et al., 2008)	HC	RRMS: increased	
			SPMS: increased	
Sorbitol	CSF (Regenold et al., 2008)	HC	RRMS: increased	
			SPMS: increased	
Mannose	CSF (Reinke et al., 2014)	Non-MS controls	MS: decreased	
**Others**				
Phosphate	Plasma (Poddighe et al., 2017)	HC	MS: decreased	
Choline	CSF (Reinke et al., 2014)	Non-MS controls	MS: increased	
	Serum (Cocco et al., 2015)	HC	MS: increased	
Trimethylamine N-oxide	Urine (Gebregiworgis et al., 2016)	HC	MS: increased	
Acetate	CSF (Kim et al., 2017)	HC and NMO	MS: decreased	
	Serum (Moussallieh et al., 2014)	NMO	MS: decreased	
	CSF (Sinclair et al., 2010)	IIH and “other diseases”	MS: increased	
Vitamin E	Serum (Salemi et al., 2010)	HC	MS: decreased	
Citrate	CSF (Kim et al., 2017)	HC	MS: decreased	
	CSF (Sinclair et al., 2010)	IIH and “other diseases”	MS: decreased	
	CSF (Reinke et al., 2014)	Non-MS controls	MS: decreased	
Oxaloacetate	CSF (Sinclair et al., 2010)	IIH and “other diseases”	MS: decreased	
**Amyloid**				
ABX-38	CSF (Augutis et al., 2013)	HC	RRMS: decreased	
			SPMS: decreased	
ABX-40	CSF (Augutis et al., 2013)	HC	RRMS: decreased	
			SPMS: decreased	
ABX-42	CSF (Augutis et al., 2013)	HC	RRMS: decreased	
			SPMS: decreased	
a-sAPP	CSF (Augutis et al., 2013)	HC	RRMS: decreased	
			SPMS: decreased	
b-sAPP	CSF (Augutis et al., 2013)	HC	RRMS: decreased	
			SPMS: decreased	
Glycolic acid—classification uncertain	CSF (Park et al., 2016)	HC	MS: increased	
Polyol—classification uncertain		RRMS	SPMS: increased	
2-Aminobutyrate (a-aminobutyric acid)	CSF (Sinclair et al., 2010)	IIH and “other diseases”	MS: increased	
1,3-Dimethylurate (dimethyluric acid)	CSF (Sinclair et al., 2010)	IIH and “other diseases”	MS: increased	
IgG	CSF (Lim et al., 2017)	HC	MS: increased	
Inosine	CSF (Park et al., 2016)	HC	MS: increased	
Butane-2 3-diol	CSF (Park et al., 2016)	HC	MS: increased	
2-Hydroxypyridine	CSF (Park et al., 2016)	HC	MS: increased	HC < remitting MS < relapsing MS
3-Hydroxybutyrate (b-hydroxybutyric acid)	CSF (Park et al., 2016)	HC	MS: increased	HC < remitting MS < relapsing MS
	Serum (Cocco et al., 2015)	HC	MS: increased	
	Serum (Dickens et al., 2014)	RRMS	SPMS: increased	
	CSF (Reinke et al., 2014)	Non-MS controls	MS: decreased	
	CSF (Sinclair et al., 2010)	IIH and “other diseases”	MS: decreased	
	Urine (Gebregiworgis et al., 2016)	HC	MS: decreased	
2-Hydroxybutyrate (a-hydroxybutyric acid)	CSF (Kim et al., 2017)	HC	MS: increased	
Formate	CSF (Kim et al., 2017)	HC	MS: increased	
2-Hydroxyisovalerate (2-hydroxyisovaleric acid)	CSF (Reinke et al., 2014)	Non-MS controls	MS: decreased	
3-Hydroxyisovalerate (3-hydroxyisovaleric acid)	Urine (Gebregiworgis et al., 2016)	HC	MS: increased	
Creatinine	Urine (Gebregiworgis et al., 2016)	HC	MS: decreased	
Hippurate (hippuric acid)	Urine (Gebregiworgis et al., 2016)	HC	MS: increased	
Malonate (malonic acid)	Urine (Gebregiworgis et al., 2016)	HC	MS: increased	
Methylmalonate (methylmalonic acid)	Urine (Gebregiworgis et al., 2016)	HC	MS: decreased	

### Methodological Quality Assessment

The QUADOMICS tool was developed to assess the methodologies of “-omics”-based research papers and was used to assess the methodologies of the papers included in this review (Lumbreras et al., 2008). The QUADOMICS tool is a set of 16 criteria that has been developed to rate the quality of -omics-based studies. The tool, which can provide an overall numeric rating, is an adaptation of the widely used QUADAS tool—an evidence-based quality assessment tool used in systematic reviews of diagnostic accuracy studies—supplemented with additional criteria to check the collection and handling of the differing biological samples that are used in -omics-based research. LP and AS independently assessed each paper using the QUADOMICS tool, settling disputes through consensus. EL and KJ scored question 10 of the tool for each paper. If the samples were taken from a biobank, then question 6 of the tool was marked positively. An overall score has not been provided, but the results can be interpreted from Table 3.

**TABLE 3:** Results from the quality appraisal tool QUADOMICS (Lumbreras et al., 2008).

Author/paper	1	2	3	4.1	4.2	5	6	7	8	9	10	11	12	13	14	15	16
Aasly (Aasly et al., 1997)	N	Y	Y	Y	Y	Y	U	Y	Y	Y	Y	Y	U	Y	Y	N	U
Aeinehband (Aeinehband et al., 2016)	N	Y	U	Y	Y	Y	Y	Y	Y	Y	Y	Y	U	Y	Y	Y	U
Augutis (Augutis et al., 2013)	N	Y	Y	Y	Y	Y	Y	Y	Y	Y	Y	Y	Y	Y	Y	Y	Y
Bystricka (Bystricka et al., 2017)	N	Y	N	Y	Y	Y	Y	Y	Y	Y	Y	Y	Y	Y	Y	N	U
Checa (Checa et al., 2015)	N	Y	Y	Y	Y	Y	Y	Y	Y	Y	Y	Y	U	Y	Y	N	U
Del Boccio (Del Boccio et al., 2011)	N	Y	Y	Y	Y	Y	Y	U	Y	U	Y	N	U	Y	Y	Y	U
Dickens (Dickens et al., 2014)	Y	Y	Y	Y	Y	Y	Y	Y	Y	Y	Y	Y	U	Y	Y	Y	Y
Cocco (Cocco et al., 2015)	N	Y	Y	Y	Y	Y	Y	Y	Y	Y	Y	Y	U	Y	Y	Y	U
Gebregiworgis (Gebregiworgis et al., 2016)	N	Y	Y	Y	Y	Y	Y	Y	Y	Y	Y	Y	U	Y	Y	Y	U
Hon (Hon et al., 2009b)	N	Y	Y	Y	Y	Y	Y	Y	Y	Y	Y	N	U	Y	Y	Y	U
Hon (Hon et al., 2011)	N	Y	Y	Y	Y	Y	Y	Y	Y	Y	Y	N	U	Y	Y	Y	U
Hon (Hon et al., 2009a)	N	Y	Y	Y	Y	Y	Y	Y	Y	Y	Y	N	U	Y	Y	Y	U
Kim (Kim et al., 2017)	N	Y	Y	Y	Y	Y	Y	Y	Y	Y	Y	Y	U	Y	Y	N	U
Lazzarino (Lazzarino et al., 2010)	N	Y	Y	Y	Y	Y	Y	Y	Y	Y	N	U	U	Y	Y	N	U
Lim (Lim et al., 2017)	N	Y	N	Y	Y	Y	Y	Y	Y	Y	Y	N	Y	Y	Y	N	U
Lotsch (Lotsch et al., 2017)	N	Y	Y	Y	Y	Y	Y	Y	Y	Y	Y	N	U	Y	Y	U	U
Moussallieh (Moussallieh et al., 2014)	N	Y	Y	Y	Y	Y	Y	Y	Y	Y	Y	Y	U	Y	Y	N	U
Moyano (Moyano et al., 2013)	N	Y	Y	Y	Y	Y	Y	Y	Y	Y	Y	Y	U	Y	Y	N	U
Narayanaswamy (Narayanaswamy et al., 2015)	N	Y	Y	Y	Y	Y	U	U	Y	Y	Y	N	U	Y	Y	N	U
Navarro and Segura. (Navarro and Segura, 1988)	N	Y	Y	Y	Y	Y	U	U	Y	Y	Y	N	U	Y	Y	N	U
Park (Park et al., 2016)	N	Y	Y	Y	N	Y	Y	Y	Y	Y	Y	Y	U	Y	Y	N	Y
Pieragostino (Pieragostino et al., 2015)	N	Y	Y	Y	Y	Y	Y	Y	Y	Y	Y	Y	U	Y	Y	Y	U
Poddighe (Poddighe et al., 2017)	N	Y	Y	Y	Y	Y	Y	Y	Y	Y	Y	Y	U	Y	Y	N	U
Pruss (Pruss et al., 2013)	N	Y	Y	Y	Y	Y	Y	Y	Y	Y	Y	Y	Y	Y	Y	N	U
Regenold (Regenold et al., 2008)	N	Y	Y	Y	Y	Y	Y	Y	Y	Y	Y	U	U	Y	Y	N	U
Reinke (Reinke et al., 2014)	N	Y	Y	Y	Y	Y	Y	Y	Y	Y	U	Y	U	Y	Y	N	U
Salemi (Salemi et al., 2010)	N	Y	Y	N	N	N	Y	Y	Y	Y	N	Y	U	Y	Y	N	U
Sinclair (Sinclair et al., 2010)	N	Y	Y	Y	Y	Y	Y	Y	Y	Y	Y	Y	U	Y	Y	Y	U
Sternberg (Sternberg et al., 2011)	N	Y	Y	Y	Y	Y	U	Y	Y	Y	Y	U	U	Y	Y	N	U
Vergara (Vergara et al., 2015)	N	Y	Y	Y	Y	Y	Y	Y	Y	Y	Y	Y	U	Y	Y	N	U

*Y, yes; N, no U, unclear. Refer to Additional File 1 for the key to the questions asked in the QUADOMICS tool. Additional File 1 Doc. Key to the questions included in the QUADOMICS (Lumbreras et al., 2008) tool. The data is the 10 questions that the QUADOMICS tool refers to. To ensure the table was formatted cleanly, the key is presented in an additional file.*

## Results

**FIGURE 1:**
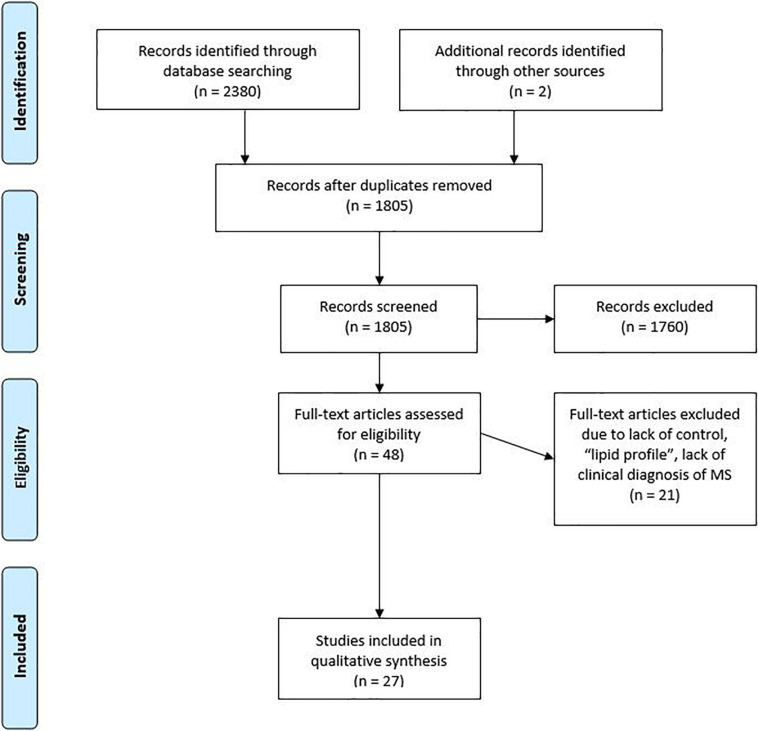
Flowchart showing the steps at which studies were excluded, following PRISMA guidelines.

A total of 1805 studies were recorded following the literature search (Figure 1). 1760 of those studies were removed based on their abstract not meeting the inclusion criteria. The full text was reviewed for 48 studies of which 27 were considered relevant for inclusion in the systematic review. The main reason that studies were excluded from analysis was that they focused on the “lipid profile,” they lacked a control, or the aim of the study was to discover the impact of an intervention.

The 27 included studies reported data on potential biomarkers that could potentially be used to discriminate between the various subtypes of MS and healthy controls. While no limitations were placed on date of publication, 18 of the 27 studies were published within the past five years. The most common type of study was case–control (26 studies), while some studies employed longitudinal data to track the change in metabolites over a course of time. Eight studies included people with other neurological disorders (OND), CIS, and/or Neuromyelitis Optica (NMO). Not all the studies recruited healthy controls, and many studies did not distinguish between the different phenotypes of MS. Some longitudinal studies used baseline values as a comparator, while some cross-sectional studies used samples of people with other conditions as a control. The most common technique used in the studies was nuclear magnetic spectroscopy, followed by liquid or gas chromatography coupled with various detectors.

Many of the studies presented metabolites that were not replicated in another study. The studies that did report on repeated metabolites often produced conflicting results.

### Fatty Acids and Lipid Metabolism Derivatives

A total of 15 of the shortlisted papers described 49 different fatty acids and lipid metabolite derivatives across CSF, venous blood, serum, plasma, and urine samples. Fatty acids are the building blocks of lipids and act as important sources of energy and as structure for cells and act on biological processes. Fatty acid levels have been shown to be altered in different diseases and may potentially act as biomarkers for polycystic ovary syndrome (Zhengao et al., 2019).

Six studies (Pitt et al., 2000; Sinclair et al., 2010; Hon et al., 2011; Dickens et al., 2014; Housley et al., 2015) reported on levels of 3-hydroxybutyrate in people with MS. The results were split with three studies (Pitt et al., 2000; Dickens et al., 2014) reporting an increase in the metabolite, while the other three reported a decrease. Cocco et al. (2015) reported an increase in the metabolite in the serum of people with MS compared to healthy controls while Park et al. (2016) reported a similar result in CSF. Dickens et al. (2014) reported an increase in the metabolite in the serum of people with SPMS when compared to people with RRMS. Reinke et al. (2014) and Sinclair et al. (2010) both reported a decrease in the metabolite in CSF of people with MS when compared to non-MS controls (Housley et al., 2015) and in people with IIH and “other diseases” (Sinclair et al., 2010). Gebregiworgis et al. (2016) reported a decrease in 3-hydroxybutyrate in the urine of people with MS when compared to healthy controls.

### Amino Acid Metabolism and Derivatives

Amino acids are the building blocks of proteins and other nitrogen-containing compounds such as creatinine, peptide hormones, nucleotides, and some neurotransmitters. The human body needs 20 different amino acids to function properly, with the body creating 11 of these (non-essential amino acids). The other 9 essential amino acids must be obtained through external sources (i.e., diet).

Four studies reported on the concentration of L-glutamate (non-essential amino acid) in people with MS. One study Poddighe et al. (2017) found that levels of L-glutamate in plasma increased in people with MS when compared to HC, while another study (Pieragostino et al., 2015) reported that glutamate increased in the CSF of people with MS compared to HC. Similarly, Moussallieh et al. (2014) found that CSF concentration of L-glutamate increased in MS when compared to people with idiopathic intracranial hypertension (IIH) and “other diseases.” The study by Moussallieh et al. (2014) discovered that people with MS had decreased levels of L-glutamate in their serum when compared to people with NMO. Two studies (Tisell et al., 2013; Moussallieh et al., 2014) used spectroscopy while (Pieragostino et al., 2015) used gas chromatography/mass spectrometry (GC/MS) and (Checa et al., 2015) used MALDI-TOF mass spectrometry.

Three studies (Tisell et al., 2013; Davis and Liu, 2015; Poddighe et al., 2017) measured the change in L-glutamine between HC and people with MS. The study by Aasly et al. (1997) determined a decrease in L-glutamine in the CSF of people with MS when compared to the CSF of HC and found that the levels in people with RRMS decreased when compared to people with chronic progressive MS. The study by Moussallieh et al. (2014) found that there was a decrease of L-glutamine in the serum of people with MS when compared to HC and an increase when compared to people with NMO. The study Poddighe et al. (2017) reported that in plasma, people with MS had increased levels of L-glutamine when compared to HC.

Two studies (Chen and Guillemin, 2009; Lim et al., 2017) reported on the ratio of quinolinic acid (QA)/kynurenic acid (KA) in MS. The study by Aeinehband et al. (2016) found the ratio in CSF increased in people undergoing a relapsing stage of RRMS when compared to people experiencing a remission in RRMS. Similarly, Lim et al. (2017) reported that the ratio of QA/KA in serum increased in people with PPMS and SPMS when compared to healthy controls. QA and KA metabolites are both downstream metabolites of the amino acid tryptophan (essential amino acid).

### Carbohydrate Metabolism and Derivatives

Carbohydrates are the primary energy source for the body and are preferentially used by the central nervous system. Carbohydrates are broken down to monosaccharides such as glucose and stored in the body as the polysaccharide, glycogen.

Three studies (Pitt et al., 2000; Yelamanchi et al., 2016; Kim et al., 2017) reported statistically significant results on the level of glucose in people with MS. Two studies (Pitt et al., 2000; Yelamanchi et al., 2016) reported levels of glucose in serum while another study (Kim et al., 2017) reported levels in CSF. All studies reported a decrease of glucose in people with MS when compared to their controls. The study by Dickens et al. (2014) found that people with SPMS had decreased levels of glucose compared to people with RRMS. The study by Kim et al. (2017) found that glucose decreased in people with MS when compared to healthy controls and people with NMO.

### Other

Other types of molecules that this review highlighted include ions which enable the flow of electrical signals through the body as well as regulating the osmotic pressure in cells and help maintain the function of muscles and nerve cells. The review also included a study of the fat soluble, Vitamin E (Salemi et al., 2010). The study recorded a decrease in the vitamin in people with MS compared to healthy controls. Vitamin E has many uses in the body including acting as an antioxidant and to boost the immune system.

Three studies (Sinclair et al., 2010; Housley et al., 2015; Kim et al., 2017) all reported a decrease in citrate in the CSF of people with MS. The study by Kim et al. (2017) compared people with MS to healthy controls while Sinclair et al. (2010) compared people with MS to people with IIH and “other diseases,” and Reinke et al. (2014) compared people with MS to non-MS controls.

#### Quadomics Assessment

The QUADOMICS tool was developed in response to a need to assess studies using “-omics”-based technologies. The selected studies had relatively similar results when assessed by the QUADOMICS tool. Areas where the studies failed to give enough information were as follows: clearly describing the selection criteria, describing in enough detail the execution of the reference standard to permit replication, stating whether the study was blinded, reporting uninterpretable/intermediate test results, and describing whether the presence of overfitting was avoided. An overall score was purposely left absent for each study.

## Discussion

A metabolomic biomarker would revolutionize how MS is diagnosed allowing patients to be diagnosed earlier potentially improving their prognosis as lifestyle changes and disease modifying therapies could be started earlier, reducing the extent of neurological damage (Noyes and Weinstock-Guttman, 2013). Metabolomic biomarkers may also give a clearer indication of disease progression and treatment efficacy, allowing treatments to be changed if they are not effective.

This systematic review assessed studies that sought to find biomarkers capable of predicting MS disease onset and/or progression. Using our comprehensive search criteria, the number of biomarkers identified as being relevant to MS was extensive. Twenty eight papers met pre-set criteria for inclusion in the study. The heterogeneity of study designs and outcome measures limited analysis to a narrative synthesis. Unfortunately, very few studies shared similar outcomes when they measured the same metabolite. This could be due to MS disease heterogeneity as well as previously mentioned technical and chemometric limitations, variability in the sample cohorts, and/or differences in the classification criteria. A clear example of this was the metabolite beta-hydroxybutyrate, a small ketone body derived from fatty acid oxidation (Newman and Verdin, 2014). Six studies published data on this metabolite, half suggesting it increased in people with MS and the other half suggesting it decreased.

Eight studies compared people with MS to people with other neurological diseases such as NMO and CIS. While these studies did not always contain healthy controls, reducing the power of their findings, their results are important as current markers of disease are general inflammatory markers and not specific to MS. Metabolites that are discussed below were studied by more than one group.

Glutamate is a non-essential amino acid and has multiple functions in the body. Glutamate is an excitatory neurotransmitter in the central nervous system and acts as a precursor molecule for the synthesis of other metabolites and as a substrate in the synthesis of amino acids (Yelamanchi et al., 2016). The study by Sinclair et al. (2010) found that the concentration of L-glutamate in CSF was increased in MS when compared to people with IIH and “other diseases,” while Moussallieh et al. (2014) found that people with NMO had increased levels of glutamate when compared to people with MS. These results where replicated in the CSF of people with MS compared to healthy controls (Checa et al., 2015) and in the serum of people with MS compared to healthy controls (Pieragostino et al., 2015). It is not clear from these studies whether the levels of glutamate are similar in people with IIH, NMO, and healthy controls as parameters were different in each study. These data suggest that glutamate could have the potential to differentiate people with MS from other neurological, inflammatory conditions. Studies have investigated glutamate excitotoxicity contributing to lesions characteristic of MS in an animal model of MS and have inferred that it may be an important mechanism in human autoimmune demyelination (Pitt et al., 2000; Matute et al., 2001). The increase in glutamate is also consistent with findings by Srinivasan and Tisell who found that glutamate concentrations were higher in MRI scans of normal-appearing white matter in MS patients compared to healthy controls (Srinivasan et al., 2005; Tisell et al., 2013).

The precursor and main source for glutamate in the brain is glutamine (Tapiero et al., 2002). The three studies that measured glutamine produced variable results; however, this could be because three different mediums were used to measure the metabolite. Levels of glutamine were decreased in CSF (Hon et al., 2011) possibly due to an increase in glutamate consuming its precursor. As with other results, follow-up studies will need to be conducted to determine whether glutamine/glutamate metabolism can be used to produce a reliable biomarker of disease.

All three studies that published data on the concentration of glucose in people with MS reported a decrease when compared to healthy controls and people with NMO. It has been theorized that glucose metabolism is affected in people with MS (Mathur et al., 2014). Perturbed glucose metabolism has been discovered in other neurological disorders including Alzheimer’s, Parkinson’s, and Huntington’s diseases (Mathur et al., 2014). Alterations in the levels of citrate in people with MS compared to HC is further evidence that glucose metabolism could be perturbed in people with MS. All three studies reported that people with MS had a decrease in citrate.

The differences in glutamate and glutamine may also be related to the results involving the kynurenine pathway. The kynurenine pathway is the metabolic path of tryptophan to produce nicotinamide adenine dinucleotide (NAD) (Davis and Liu, 2015). NAD is a vital cofactor that regulates glucose metabolism acting as an electron transfer molecule in the electron transport chain. Given the recent interest in NAD and its link with aging and metabolic disease, especially as described by Chini et al., it may be worthwhile to further explore the role of NAD both in the treatment and pathogenesis of MS (Verdin, 2015).

By-products of the pathway include quinolinic acid (QA) and kynurenic acid (KA). QA is an amino acid that activates the NMDA receptor on excitatory neurons, causing the neurotransmitter glutamate to be released and uptake inhibited (Chen and Guillemin, 2009). KA is a NMDA receptor antagonist, blocking the excitotoxic effects of QA (Lim et al., 2017). KA also has antioxidant effects and is neuroprotective (Lim et al., 2017). Studies by Aeinehband et al. (2016) reported alterations in the ratio of QA/KA in people with MS, indicating abnormal kynurenine pathway metabolism in people with MS. Aeinehband reported an increase in the ratio in CSF of people with RRMS who were experiencing a relapsing phase, compared to people in a remitting phase. Lim found that the ratio of QA/KA increased in the serum people with PPMS and SPMS compared to healthy controls and that people with RRMS had higher levels of KA. These data, together with findings by Cocco et al. (2015) that suggest decreased levels of tryptophan in the serum of people with MS, indicate an increase in the activity of the kynurenine pathway in people with MS.

Three studies (Chen and Guillemin, 2009; Cocco et al., 2015; Lim et al., 2017) used multiple cohorts to validate their results. The first cohort of samples was used to measure differences in metabolic profiles between people with MS and healthy controls so that a model could be developed to test in the second cohort. Sinclair et al. (2010) reported that the model generated from the first cohort identified patients with MS in the second cohort with 67% sensitivity and 75% specificity. Lim et al. (2017) used six predictors, with QA and KA being the most important, to develop a model that had a prediction accuracy of 83% when validating HC, RRMS, and SPMS from another cohort. The kynurenine pathway also is altered in patients with systemic lupus erythematosus (SLE) and associated with severe fatigue (Akesson et al., 2018), limiting its ability to differentiate MS from people with other autoimmune diseases.

The study by Dickens et al. (2014) produced a model that differentiated people with RRMS from people with SPMS using metabolomics and partial least squares discriminant analysis (PLS-DA) of biofluids. Unfortunately, due to the small sample size, the model was not able to differentiate people with SPMS from PPMS. While the sample sizes were small in these studies, the idea of generating a model from multiple differences in metabolites has merit and should be explored further.

### Strengths and Limitations

The strength of this review is that it used comprehensive search criteria, resulting in a large sample of studies to be reviewed. The studies included in this review all include patients with clinically confirmed MS; however, the exclusion criteria could have been more clearly defined. Due to the small sample sizes and limited follow-up studies, the power of this review was diminished and recommendations based on inferential statistics cannot be made. While every study featured patients clinically diagnosed with MS, the diagnosis classification used varied between studies and many did not differentiate between the different phenotypes of MS. Furthermore, our analysis did not factor the participants’ age and gender into the analysis. In addition, many studies did not stand up to the rigor of the QUADOMICS tool, particularly those where samples were taken from biobanks, as not enough information was provided. This could be explained due to the fact that many of the studies assessed were not necessarily diagnostic, which is what the tool was designed for. In retrospect, the tool could have been modified to suit the types of studies that were assessed. It is advised that future “-omics”-based studies keep the QUADOMICS tool in mind when conducting and publishing studies as a few small inclusions would have resulted in more positive scores on the test. A considerable limitation was the exclusion of studies that focused on the “lipid profile.” This was considered due to the large number of studies that analyzed the basic lipid profile, which we defined as cholesterol (both high-density lipoprotein and low-density lipoprotein) and triglycerides. It is intended that a follow-up study will be completed that focuses on studies involving the lipid profile.

## Conclusion

Several promising candidate biomarkers suitable for clinical application in MS have been studied.

The studies that showed the most promise were those pertaining to glucose metabolism, glutamate, and tryptophan metabolism, specifically the kynurenine pathway. Kynurenine pathway metabolites have been identified as possible biomarkers for inflammatory diseases and neurodegenerative diseases such as amyotrophic lateral sclerosis (ALS) and Alzheimer’s, Parkinson’s, and Huntington’s diseases (Nemeth and Vecsei, 2006). QA could potentially be used to not only assist diagnosis of MS but also subtype it. The investigation by Lim et al. (2017) found that QA was found in higher concentrations in PPMS compared with SPMS which was higher than RRMS. Furthermore, analyzing the ratio between KA/QA enabled the team, with a high degree of certainty, to model and predict different subtypes of MS with a high sensitivity.

It is recommended that a large age- and gender-matched study that compares the different phenotypes of MS (RRMS, SPMS, and PPMS) to a healthy control utilizing an easily accessible fluid such as serum should be carried out focusing primarily on glucose metabolism, and the kynurenine pathway to validate the results of Lim et al. (2017)

While a single metabolite that could act as a biomarker of disease status is the ideal scenario, a model generated from multiple biomarkers involved in a variety of metabolomic processes may be the best possibility for a conclusive diagnostic test for MS. The identification of a biomarker, or a model from multiple metabolomic biomarkers, that can correctly diagnose MS and determine response to treatment may result in better outcomes for people diagnosed with this debilitating disease.

## Author Contributions

GJ, TW, AL, CB, and LP conceived the project. LP drafted the manuscript. TW supervised the lead author LP and AS and provided extensive comments on drafts. LP and AS undertook abstract reviews, full-text reviews, and quality appraisals. CB screened full-length manuscript for relevance. CL and KJ undertook quality appraisal of technical areas (Quadomics tool item 10). All authors contributed to editing and approved the final manuscript.

## Conflict of Interest

The authors declare that the research was conducted in the absence of any commercial or financial relationships that could be construed as a potential conflict of interest.

## Abbreviations

MS: Multiple Sclerosis
RRMS: Relapsing Remitting MS
SPMS: Secondary Progressive MS
PPMS: Primary Progressive MS
CIS: Clinically Isolated Syndrome
OCB: Oligoclonal Bands
CSF: Cerebrospinal Fluid
NMR: Nuclear Magnetic Resonance
MeSH: Medical Subject Heading
OND: Other Neurological Disorder
NMO: Neuromyelitis Optica
HC: Healthy Control
IIH: Idiopathic Intracranial Hypertension
QA: Quinolinic Acid
KA: Kynurenic Acid
PLS-DA: Partial Least Squares Discriminant Analysis
SLE: Systemic Lupus Erythematosus.
